# Decision Support for Personalized Cloud Service Selection through Multi-Attribute Trustworthiness Evaluation

**DOI:** 10.1371/journal.pone.0097762

**Published:** 2014-06-27

**Authors:** Shuai Ding, Chen-Yi Xia, Kai-Le Zhou, Shan-Lin Yang, Jennifer S. Shang

**Affiliations:** 1 School of Management, Hefei University of Technology, Hefei, P.R. China; 2 Key Laboratory of Process Optimization and Intelligent Decision-Making, Ministry of Education, Hefei, P.R. China; 3 Tianjin Key Laboratory of Intelligence Computing and Novel Software Technology and Key Laboratory of Computer Vision and System (Ministry of Education), Tianjin University of Technology, Tianjin, P.R. China; 4 The Joseph M. Katz Graduate School of Business, University of Pittsburgh, Pittsburgh, Pennsylvania, United States of America; Semmelweis University, Hungary

## Abstract

Facing a customer market with rising demands for cloud service dependability and security, trustworthiness evaluation techniques are becoming essential to cloud service selection. But these methods are out of the reach to most customers as they require considerable expertise. Additionally, since the cloud service evaluation is often a costly and time-consuming process, it is not practical to measure trustworthy attributes of all candidates for each customer. Many existing models cannot easily deal with cloud services which have very few historical records. In this paper, we propose a novel service selection approach in which the missing value prediction and the multi-attribute trustworthiness evaluation are commonly taken into account. By simply collecting limited historical records, the current approach is able to support the personalized trustworthy service selection. The experimental results also show that our approach performs much better than other competing ones with respect to the customer preference and expectation in trustworthiness assessment.

## Introduction

Cloud computing has become the driver for innovation in the recent years, from startups (e.g. Dropbox, Instagram) to established enterprises (Samsung). They are all using cloud computing to better serve their customers around the world [1]. Cloud service is also gaining wide acceptance and becoming popular to individuals as it reduces hardware and licensing costs, and it is scalable and allows users to work from any computer anywhere.

Several leading IT enterprises including Google, IBM, Microsoft, and Amazon have started to offer cloud services to their customers [2]–[4]. While many small and medium-sized enterprises (SMEs) and individual customers prefer to apply cloud services to build their business system or personal applications, they are often facing two major challenges at the selection time: (1) multiple cloud services are often available by different venders providing similar functional properties (i.e., “functionally-equivalent”). Customers usually lack appropriate, qualified, sufficient information and benchmarks to assess cloud services with regard to individual preferences and market dynamics [5]; (2) although cloud service vendors are struggling to improve service quality and performance, cloud computing are not necessarily trustworthy – unhandled exceptions and crashes may cause cloud service to deviate dramatically from the expectation [6], [7]. Therefore, there is an increasing demand to help the non-expert customers with the selection of trustworthy cloud service.

The trustworthiness of cloud service affects customers' perception towards service quality, which has significant bearing on customer satisfaction and royalty. The trustworthy attributes include reliability, scalability, availability, safety, security, etc [8]–[10]. Designing a general and comprehensive analytical model for trustworthiness evaluation is challenging, as the model needs the assessor to achieve, in reasonable time, useful results to determine the best service option. Due to their commercial value (similar to online recommendation system), several evaluation models [11]–[14] have been proposed by academia and industry lately. These models focus on quantitative analysis and evaluate trustworthiness through a collectively exhaustive dataset.

Except for some specific cases, the assessment dataset remains very sparse due to the costly and time-consuming nature of cloud service invocation. Intuitively, without sufficient data, fair review of cloud services cannot be achieved by existing evaluation methods [9], [15]. Fortunately, cloud vendors can collect historical records (QoS values, customer ratings, etc) from different cloud applications in cloud computing environment. With the vast amount of collaborative filtering (CF) technologies available in the field of online recommendation system, we believe there is a strong theoretical foundation to derive a generic trustworthiness model to support the evaluation of cloud service.

There have been some attempts to improve the accuracy of cloud service assessment by a CF process. However, very little attention is paid to the trustworthiness of cloud service, and no interest is given to the case when significant attribute values are missing. The lack of general and formal methodology can be attributed to the large process gap between the cloud service recommenders and trustworthiness researchers. To deal with this challenge, we propose a new CF approach to make use of hidden information (i.e. experience usability, value distribution) to measure the similarity between different services. Moreover, to support personalized selection of cloud services, we also provide a natural treatment for multi-attribute aggregation taking into account customer's preference and expectation.

## Background

In the current market, multiple cloud services of similar functions are often available for specific domains. For example, in cloud storage service (e.g. data service, online file system, online backup plan), over 100 functionally-equivalent cloud services are offered by vendors. Some typical examples can be found in Table 1. Given the lack of cloud computing experience of non-expert customers, it is tedious to manually select an appropriate candidate from a set of functionally-equivalent services. Therefore, cloud service evaluation through quality analysis has gained much attraction among service-oriented computing and cloud computing communities over the past two decades.

**Table 1:** Online cloud storage services.

Vender	Cloud Service	Feature	Pricing
Amazon	EBS	Storage Service	$0.1 per GB-month, $0.1 per 1 million I/O requests
Amazon	S3 Standard	Storage Service	$0.095 per GB-month, $0.005 per 1000 requests
Google	Google Cloud Storage	Storage Service	$0.085 per GB-month, $0.01 per 1000 ops-month
IBM	SoftLayer Object Storage	Storage Service	$0.1 per GB-month
Microsoft	Azure Data Service	Storage Service	$0.095 per GB-month, $0.01 per 100000 I/O requests
Apple	iCloud	Storage Service	$20 for 10 GB upgrade
GoGrid	GoGrid Cloud Storage	Storage Service	$0.12 per GB-month
JustCloud	JustCloud Cloud Storage	Storage Service	$3.95 per month, unlimited storage
ZipCloud	ZipCloud Online Storage	Storage Service	$6.95 per month, unlimited storage
AT&T	Synaptic Storage	Storage Service	Unknown
LiveDrive	Livedrive Backup Plan	Backup System	$6 per month, 2 TB storage space
CrashPlan	CrashPlan Backup Plan	Backup System	$5.99 per month, unlimited storage
Carbonite	Cloud Backup Services	Backup System	$59.99 per year, unlimited storage
FlexiScale	FlexiScale Public Cloud	Platform Service	$17 per 1000 unit-hour
AppNexus	AppNexus Cloud	Platform Service	Unknown
Rackspace	Mosso cloud files	File System	$0.75 per GB-month
HighTail	HighTail	File System	$15.99 per month, unlimited storage
Amazon	SimpleDB	Database	$0.12 per GB-month

Given the intricate interactions among QoS (Quality of Service) attributes, customer preferences and market dynamics that jointly influence the perceived quality of cloud services, developing a market-relevant analytical model is crucial to cloud service selection [16]–[18]. Due to their commercial value and the associated research challenges, many researchers and practitioners have studied the topics. Two types of service selection models are widely examined: evaluation-focused service selection models and prediction-focused service selection models.

By achieving market-relevant evaluations, customers can identify risks and benefits of each cloud service application and choose the best for adoption. The most employed evaluation models include: AHP-based cloud service ranking [19], reputation-aware service rating [20], trust-aware service selection [21], brokerage-based selection [22], SLA-based cloud trustworthiness estimation [11], trustworthy service selection [23]. Although these techniques can accurately and exhaustively estimate service quality, their implementation is time-consuming and costly.

Instead of real-world cloud service invocations, the prediction-focused service selection models can produce QoS values or service ranking using collaborative filtering (CF). The CF approaches for cloud service selection can be categorized as: item-based approaches [24], customer-based approaches [25], their fusion approaches [26], model-based approaches [27], and ranking-oriented approaches [28], where the first three categories are rating-oriented approaches. These approaches help assessors predict the missing attribute values by exploiting neighbors' usage experiences. Several collaborative filtering approaches for cloud service selection have been studied, but they did not consider customer preference and expectation in trustworthiness assessment.

In the prediction process, similar neighbors (customers or services) are identified to generate useful collaborative information. Popular choices for similarity estimation include Pearson correlation coefficient (PCC) [29] and vector similarity (VS) [30]. Since these measures only consider the numerical relationship between different ratings, they remain imprecise and confusing for estimating the neighbor similarity to support missing value prediction. Concerned that PCC may overestimate the similarities of negative services, Zheng et al. [26] propose a significance weight and modify PCC to improve the accuracy of similarity computation in service recommendation. However, the significance weight affects the similarity computation of positive services with more usage experiences. To address this problem, Ding et al. [31] define a convex function (usage structure factor) to reflect the usability of customer experience.

While a great number of researchers have focused on the trust-aware service selection and recommendation, little attention has been devoted to the role of customer preference and expectation in multi-attribute trustworthiness evaluation [32]. In addition, large quantities of works offer some valuable clues to discern between different services, the significances arising from value distribution is seldom considered. Thus, we will here combine evaluation-focused and prediction-focused approaches to propose a novel trustworthiness evaluation method which will fully utilize the information of similar services and customer's experience, and take into account both the missing attribute value prediction and the multi-attribute trustworthiness evaluation at the same time.

## Methods and Materials

Based on the fact that the size and rate of growth in customers outweigh the expansion of delivered services in the cloud computing market, we employ item-based CF approach rather than the user-based or their fusion approach to produce the missing attribute values in trustworthiness evaluation. Motivated by the observation that experience usability and value distribution could provide valuable insight and distinctive information in the CF process, we create a new similarity measure for enhancing the prediction performance.

### Pearson Correlation Coefficient

To make an accurate prediction, we first estimate the similarity between different cloud services. Given a service selection problem consisting of *M* customers and *N* services, the customer-service matrix for missing value prediction is denoted as
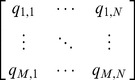
(1)where the entry *q_m,n_* denotes a historical record (QoS value or customer rating) of cloud service *cs_n_* made by customer *u_m_*, “*q_m,n_* = *null*” states that *u_m_* didn't invoke *cs_n_* yet.

#### Pearson Correlation Coefficient (PCC)

[29] Taking use of numerical distance to estimate the correlation between different services, PCC has been successfully adopted for recommendation system evaluations. Let *cs_n_* and *cs_v_* be two services, *U_n,y_* be the subset of customers who have invoked both *cs_n_* and *cs_v_*, then PCC is applied to calculate the similarity between *cs_n_* and *cs_v_* by
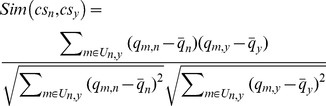
(2)where *Sim*(*cs_n_*, *cs_v_*) is in the interval of [−1, 1], 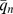 and 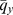 stand for the average values of *cs_n_* and *cs_v_* made by different customers. However, as noted in Ref. [26], PCC always overestimate the similarities of negative services, which are actually not similar but happen to have similar usage experience made by few customers. Table 2 shows a simple customer-service matrix which contains six customers (*u*_1_ to *u*_6_) and ten cloud services (*cs*_1_ to *cs*_10_). When utilizing Eq. (2), we calculate the PCC values between the services, and get the following relation: *Sim*(*cs*_1_, *cs*_3_)>*Sim*(*cs*_1_, *cs*_4_)>*Sim*(*cs*_1_, *cs*_2_), which indicates *cs*_3_ is more similar to *cs*_1_ than *cs*_2_ and *cs*_4_. It is clearly contrary to the reality due to the limited usage experience. Therefore, it is necessary to reinforce the similarity information in the CF process.

**Table 2:** A simple customer-service matrix.

	*cs*_1_	*cs*_2_	*cs*_3_	*cs*_4_	*cs*_5_	*cs*_6_	*cs*_7_	*cs*_8_	*cs*_9_	*cs*_10_
***u*** **_1_**	0.9	0.7	*null*	1	0.8	0.6	0.4	0.7	*null*	*null*
***u*** **_2_**	0.8	0.7	0.8	0.4	0.6	0.6	0.7	*null*	0.9	0.8
***u*** **_3_**	0.9	0.8	*null*	0.6	0.5	0.5	0.5	*null*	*null*	1
***u*** **_4_**	0.8	0.9	*null*	1	*null*	0.8	0.6	0.7	0.6	*null*
***u*** **_5_**	0.7	0.6	*null*	0.5	0.7	*null*	0.8	*null*	0.4	0.9
***u*** **_6_**	*null*	0.8	0.9	0.6	*null*	*null*	0.9	0.8	0.8	0.7

### Significance estimation

It seems logical to believe that some cloud services in customer-service matrix may have high significances in making recommendations [33], [34]. For instance, a cloud service, which has more useful historical records, may be regarded as more important compared with a negative service. PCC is only related to the numerical distance between different services, but it has nothing to do with the statistical features of historical records. For this case, we introduce two types of significances arising from the experience usability and value distribution of historical records, respectively.

#### Estimating the experience usability

To determine the significance of neighbors in a CF process, one often assumes a linear relationship between usage experiences and neighbor significances [26], [31]. One difference of our work from traditional CF approaches is that we apply a distance measurement method to estimate the experience usability in customer-service matrix. During the distance measurement, Jaccard's coefficient [35] is frequently employed to estimate the discrimination of asymmetric information on binary variables. Before integrating Jaccard's coefficient into our similarity measure, we map the original customer-service matrix into a rectangular binary matrix as follows:
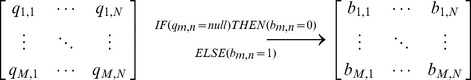
(3)where the entry *b_m,n_* = 1 denotes the customer *u_m_* has invoked the service *cs_n_* previously, whereas *b_m,n_* = 0 denotes that *u_m_* didn't invoke *cs_n_*. Let |*U_n_*| be the number of customers who has invoked *cs_n_* before, and |*U_n,y_*| be the number of customers who invoked both *cs_n_* and *cs_v_*. We use the Jaccard's coefficient J_*n,y*_ to reflect the rise of significance due to the experience usability, which can be expressed mathematically as:
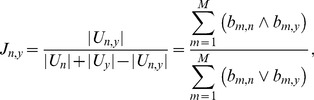
(4)where J_*n,y*_ is in the interval of [0, 1], and a larger J_*n,y*_ value indicates that the historical records made over *cs_v_* is more useful in the CF process. J_*n,y*_ = J_*y,n*_ holds for all services, which is consistent with the intuition that the similarity between *cs_v_* and *cs_n_* is only related to the subset of historical records made by the customers who have invoked both *cs_n_* and *cs_v_*.

Based on the customer-service matrix in Table 2, we get the significances arising from the experience usability for each service, as shown in Table 3. The values shown in grey are calculated for the negative service *cs*_3_. As observed from Table 2, *cs*_3_ has only been invoked twice. Consequently, his experience usability values are significantly lower than other services (e.g. *J*_1,3_<<*J*_1,2_). We can infer that integrating J_*n,y*_ into similarity measure will notably reduce the influence of negative service. It is worth noting that neither PCC nor J_*n,y*_ can distinguish between *cs*_2_ and *cs*_4_, since they do not have sufficient power to detect the crucial difference in value distributions.

**Table 3:** Significances arising from the experience usability.

	*cs*_1_	*cs*_2_	*cs*_3_	*cs*_4_	*cs*_5_	*cs*_6_	*cs*_7_	*cs*_8_	*cs*_9_
***cs*** **_2_**	0.833								
***cs*** **_3_**	0.167	0.333							
***cs*** **_4_**	0.833	1	0.333						
***cs*** **_5_**	0.8	0.667	0.2	0.667					
***cs*** **_6_**	0.8	0.667	0.2	0.667	0.6				
***cs*** **_7_**	0.833	1	0.333	1	0.667	0.667			
***cs*** **_8_**	0.333	0.5	0.25	0.5	0.167	0.4	0.5		
***cs*** **_9_**	0.5	0.667	0.5	0.667	0.333	0.333	0.667	0.4	
***cs*** **_10_**	0.5	0.667	0.5	0.667	0.6	0.333	0.667	0.167	0.6

#### Estimating the value distribution

The neighbors which have the same PCC similarity may have different value distributions. It is necessary to detect more hidden information in the customer-service matrix for significance estimation. For this case, we propose a method to discriminate neighbors' significances arising from their unique value distributions. In practice, the customer-service matrix is very sparse due to limited usage experiences. Therefore, we will ignore the historical records made by the customer *u*, where *u* 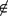 *U_n,y_*. Let *D*_n_ = {*q_m,n_* | *u_m_* 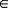 *U_n,y_*} and *D*_y_ = {*q_m,y_* | *u_m_* 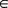 *U_n,y_*} be the historical records in similarity computation made over *cs_n_* and *cs_v_*, and |*D_y_*| be the cardinality of *D_n_*, and *dom*(*D_n_*) be the domain of *D*_n_ subject to the following constraints:
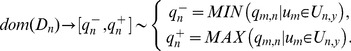
(5)Following *dom*(*D_n_*), the dataset *D*_y_ can be grouped into three categories:
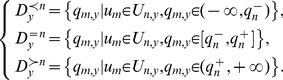
(6)Since *D_y_* is a finite discrete dataset, the probability of each category can be computed as:
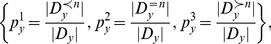
(7)where 
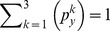
, and 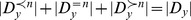. From the information entropy aspect, we use the following expression to detect the difference between the value distributions of *cs_n_* and *cs_y_*:
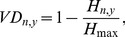
(8)where 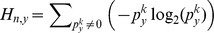 denotes the information entropy of *D_y_*, and *H_max_* denotes the maximal entropy in customer-service matrix, respectively. *VD_n,y_* is a linear function defined in [0, 1]. From the maximum entropy principle [36], we have *H_max_* = log_2_(3). Thus, Eq.(8) can be rewritten as
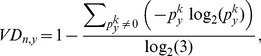
(9)where *VD_n,y_* attains its unique global minimum 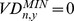 if 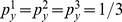; otherwise it attains global maximum 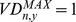 when 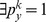.

We can thus calculate the significances *VD_n,y_* arising from the value distribution using Eq.(9) over the customer-service matrix in Table 2. Table 4 shows the values of these significances. The values shown in grey are calculated for the cloud services *cs*_2_ and *cs*_4_.

**Table 4:** Significances arising from the value distribution.

	*cs*_1_	*cs*_2_	*cs*_3_	*cs*_4_	*cs*_5_	*cs*_6_	*cs*_7_	*cs*_8_	*cs*_9_	*cs*_10_
***cs*** **_1_**		1	1	1	0.488	0.369	0.387	1	1	0.421
***cs*** **_2_**	0.545		0.685	1	1	0.488	1	0.421	1	0.488
***cs*** **_3_**	1	0.369		1	1	1	1	1	1	0.369
***cs*** **_4_**	0.387	0	1		0.054	0.054	0.079	0.421	0.488	1
***cs*** **_5_**	0.369	0.488	1	1		0.421	1	1	1	1
***cs*** **_6_**	0.488	0.488	1	1	1		0.488	1	1	1
***cs*** **_7_**	0.387	0.421	0.369	1	0.488	0.488		1	1	0.369
***cs*** **_8_**	1	1	1	1	1	1	1		1	1
***cs*** **_9_**	0.421	0.488	1	1	1	0.369	0.421	0.369		0.421
***cs*** **_10_**	0.421	0.421	1	1	1	1	1	1	0.685	

#### Similarity measurement adopting significance

After we have defined the two types of significance for each service, we can then create the similarity measure, *Sim^s^*(*cs_n_*, *cs_v_*), which takes into account the significance previously defined. To estimate the significance as accurately as possible, we identify the significance of *cs_v_* with respect to *cs_n_* as a linear combination of J_*n,y*_ and *VD_n,y_*, such that:
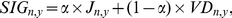
(10)where *α* is defined to determine how much our significance relies on experience usability and value distribution. If *α* = 0, we only extract the experience usability for conducting significance estimation, and if *α* = 1, we consider only the value distribution. Hence, the similarity measure can be written in standard form:
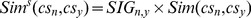
(11)where *SIG_n,y_* denotes the significance of *cs_v_* with respect to *cs_n_*, and *Sim*(*cs_n_*, *cs_v_*) denotes the PCC value between *cs_n_* and *cs_v_*. Different from existing similarity measures, our approach employs not only numerical distance but also usage experience as well as value distribution to determine the similarity between different services. With the definition of similarity measure defined in Eq.(11), for every cloud service in customer-service matrix, we rank their neighbors and select the top-*k* most similar services to make missing value prediction. Following the top-*k* similar service defined in [26], we get
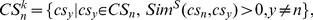
(12)where *CS_n_* denotes the neighbor set of *cs_n_* in customer-service matrix, and *Sim^S^*(*cs_n_*, *cs_v_*) denotes the similarity between *cs_n_* and *cs_v_*. For the customer-service matrix in Table 2, we set *α* to 0.8 to obtain the similarity measures between different services (see Table 5). The top 3 neighbors of each service are marked in grey areas as seen in each column.

**Table 5:** Similarities between different services.

	*cs*_1_	*cs*_2_	*cs*_3_	*cs*_4_	*cs*_5_	*cs*_6_	*cs*_7_	*cs*_8_	*cs*_9_	*cs*_10_
***cs*** **_1_**		0.361	0.334	0.366	−0.099	−0.421	−0.663	−0.24	0.402	0.141
***cs*** **_2_**	0.323		0.403	0.484	−0.379	0.376	−0.153	0	0.213	−0.081
***cs*** **_3_**	0.334	0.34		0.223	0.36	0.36	0.259	0.4	−0.165	−0.212
***cs*** **_4_**	0.314	0.387	0.223		0.336	0.303	−0.494	−0.347	−0.166	0.046
***cs*** **_5_**	−0.096	−0.326	0.36	0.453		0.324	−0.099	−0.334	−0.464	−0.432
***cs*** **_6_**	−0.435	0.376	0.36	0.408	0.39		0.145	−0.312	−0.208	−0.405
***cs*** **_7_**	−0.663	−0.135	0.189	−0.605	−0.085	0.146		0.549	0.032	−0.451
***cs*** **_8_**	−0.24	0	0.4	−0.43	−0.334	−0.312	0.549		0.518	−0.334
***cs*** **_9_**	0.324	0.184	−0.165	−0.193	−0.464	−0.152	0.027	0.393		−0.395
***cs*** **_10_**	0.14	−0.079	−0.268	0.04	−0.432	−0.405	−0.544	−0.334	−0.432	

#### Missing value prediction

With the exponential growth of cloud service on the Internet, service recommendation techniques like QoS-aware CF approaches have become increasingly important and popular [37]. Based on our similarity measure, we propose an enhanced item-based CF approach (named as JV-PCC) to reinforce the prediction performance. To predict the missing value 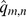 of service *cs_n_* for customer *u_m_*, we first determine the objective weight of each similar neighbor:
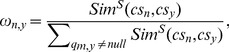
(13)where *cs_v_* 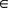 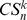 denotes a similar neighbor of *cs_n_*, while *Sim^S^*(*cs_n_*, *cs_v_*) denotes the similarity between *cs_n_* and *cs_v_*. The objective weights define the relative importance of each similar neighbor in the CF process. Next, we attain a prediction by a classic aggregation function:
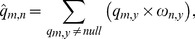
(14)where *q_m,y_* denotes the historical record of *cs_y_* made by customer *u_m_*. In practice, local runtime environment (e.g. network bandwidth) and customer's rating style may significantly influence the historical records over delivered services. However, the above function deems inappropriate as it is calculated through only one customer. To address this problem, JV-PCC predicts the missing attribute value by the following equation:
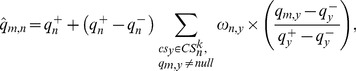
(15)where *ω_n,y_* denotes the objective weight of *cs_y_* with respect to *cs_n_*, while 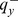 and 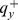 denote the minimum and maximum values of service *cs_y_*, respectively. Table 6 displays the values estimated for the missing records in Table 2. In the experimental examples, both customer-based and service-based neighborhood information were adopted for approximating the missing value.

**Table 6:** Predicted attribute values.

	*cs*_1_	*cs*_2_	*cs*_3_	*cs*_4_	*cs*_5_	*cs*_6_	*cs*_7_	*cs*_8_	*cs*_9_	*cs*_10_
***u*** **_1_**			0.843						0.609	1
***u*** **_2_**								0.808		
***u*** **_3_**			0.865					0.74	0.842	
***u*** **_4_**			0.85		0.8					0.887
***u*** **_5_**			0.831			0.593		0.76		
***u*** **_6_**	0.865				0.703	0.749				

### Trustworthiness-aware service selection

Several models, focusing on the quantitative measurement of service trustworthiness, have been proposed in Refs. [9], [18], [38]. However, different customers have different preference and expectation in service selection. A thorough understanding into these factors is essential to ensure effective evaluation finding. Here, we introduce a cloud service evaluation model, which helps aggregate trustworthy attributes by considering customer's preference and expectation.

#### Attribute utility determination

To make use of observed or estimated values, we need to know that different attributes may have inconsistent dimensions. The results in [32] show that utility can be used to identify an entity's trustworthiness. Therefore, we first derive the utility from the customer-service matrix so as to ensure their values are in the range of [0, 1]. Trustworthy attributes are often divided into quantitative and qualitative attributes, of which the former are objective measures (e.g. QoS value), and the latter are subjective customer ratings. In addition, quantitative trustworthy attributes can be grouped into two classes: “benefit” and “cost”. For “benefit” (“cost”) attribute, e.g. *throughput* (*response-time*), the higher (lower) its value is, the greater the possibility that a customer would choose it becomes. In our model, qualitative attributes are also considered as “benefit” attributes. Let *q_m,n_* be the attribute value of *cs_n_*, and the attribute utility (risk-neutral) *H_m,n_* has the following form:
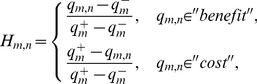
(16)where 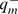 and 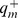 denote the minimum and maximum attribute values for customer *u_m_*, and they are subject to the following constraints:
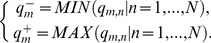
(17)The attribute utility *H_m,n_* is in the range of [0, 1], where a larger *H_m,n_* indicates that customer *u_m_* is more satisfied with the service *cs_n_*.

#### Customer satisfaction estimation

**Figure 1:**
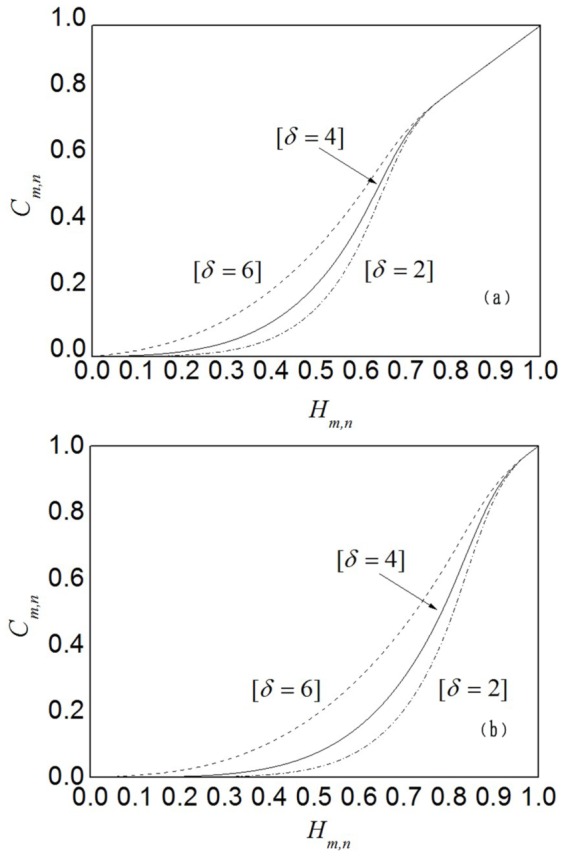
Customer satisfaction function *C_m,n_*. (a) and (b) depict the distributions of customer satisfaction as recorded at the fixed expectation *H* ^exp^ = 0.7 and *H* ^exp^ = 0.9, where the parameter *δ* is varied from 2 to 6 in increment of 2. It can be observed that the rate of change in customer satisfaction differs significantly when *H_m,n_* falls below and exceeds the expectation.

From influential theory in marketing science, we consider that the perception of cloud service trustworthiness is a customer satisfaction function, which includes customer preference and expectation attributes. In general, customer satisfaction function should exhibit two characteristics: (1) given the same expectation, a trustworthy cloud service is weighed much more heavily than an untrustworthy service. This effect is reflected in the derivation of attribute utility; (2) customer satisfaction slightly increases when attribute utility surpasses a certain value (expectation), and significantly decreases when attribute utility falls below expectation [39]. We formalize this interaction as a piecewise linear function:
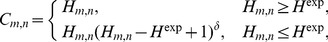
(18)where *C_m,n_* is constrained to 0≤*C_m,n_*≤1; the parameter *δ* regulates the impact of customer preference on perceived trustworthiness; and *H* ^exp^ denotes the customer expectation with regard to selecting trustworthy cloud service. As shown in Fig. 1, *C_m,n_* is continuous (i.e. the piecewise function converges at *H_m,n_ = H* ^exp^).

The severity and rate of satisfaction (controlled by *H* ^exp^ and *δ*, respectively) reflect different customer's tolerance to untrustworthy candidates. Let *H* ^exp^ = 0.7, and *δ = *2. Table 7 shows the customer satisfaction for each attribute value (historical record or predicted value), which corresponds to the original customer-service matrix in Table 2 and the predicted missing values given in Table 6.

**Table 7:** Customer satisfaction.

	*cs*_1_	*cs*_2_	*cs*_3_	*cs*_4_	*cs*_5_	*cs*_6_	*cs*_7_	*cs*_8_	*cs*_9_	*cs*_10_
***u*** **_1_**	1	0.134	0.229	1	1	0.134	0	0	0.215	1
***u*** **_2_**	0.32	0.134	0	0	0.134	0.134	0.486	1	1	0.134
***u*** **_3_**	1	0.623	0.587	0.134	0	0	0.05	0.166	0.884	1
***u*** **_4_**	0.32	1	0.32	1	1	1	0.196	0	0.196	0.531
***u*** **_5_**	0	0	0.115	0.036	0.623	0.115	0.8	0.407	0	0.623
***u*** **_6_**	0.825	0.623	1	0.134	0.645	0.83	1	0.926	0.8	0

#### Trustworthy attribute aggregation

After estimating customer satisfaction and ensuring the value of *C_m,n_* in the interval of [0, 1], the degree of trustworthiness (alias “trust value” [40]) of each cloud service in customer-service matrix can be achieved by aggregating trustworthy attributes. Let 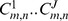 be the customer satisfaction on a set of specified attributes *A*_1_..*A*_J_, then the trust value of *cs_n_* is computed as:
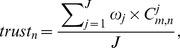
(19)where 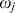 denotes the weight of trustworthy attribute *A_j_*, 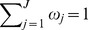.

The trust value gives the comprehensive perception of cloud service trustworthiness, while the weights modify this trust value based on the relative importance of trustworthy attributes. Actually, a set of specified trustworthy attributes can be easily weighted by applying existing technologies such as those discussed in [32]. We omit the details for brevity.

#### Decision support for personalized service selection

Multi-attribute trustworthiness evaluation is an important step for making accurate service selection. We suppose that *u_m_* is the active customer, who requires trustworthy cloud service. While the evaluation results have arrived, a set of appropriate service candidates can be identified for *u_m_* by:
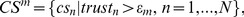
(20)where *trust_n_* denotes the trust value of *cs_n_*, 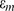 denotes the selection threshold determined by *u_m_*. We aim to remedy the shortcomings of evaluation-focused selection methods by avoiding the costly and time-consuming real-world service invocations. Note that when 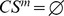 the service selection for the active customer *u_m_* needs to be degrade by decreasing the parameter 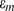.

Let 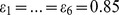, a set of trustworthy cloud services can be recommended for *u*_1_…*u*_6_ as
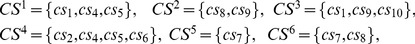
(21)where the customer satisfaction for each attribute value is presented in Table 7. In practice, our approach makes it possible to deal with various types of trustworthiness-aware cloud service selections by combing the evaluation-focused and the prediction-focused methods. Note that if trustworthiness is not the only issue that affects customer's decision making, it is necessary to extend the selection process of our approach, e.g., price-oriented service filtering, into other attributes or indexes.

## Results

In this section, abundance of experiments are conducted to show how to recommend trustworthy cloud service in the context of large sparse assessment dataset, and to verify the efficiency of our CF approach.

### Prototype implementation and results

**Figure 2:**
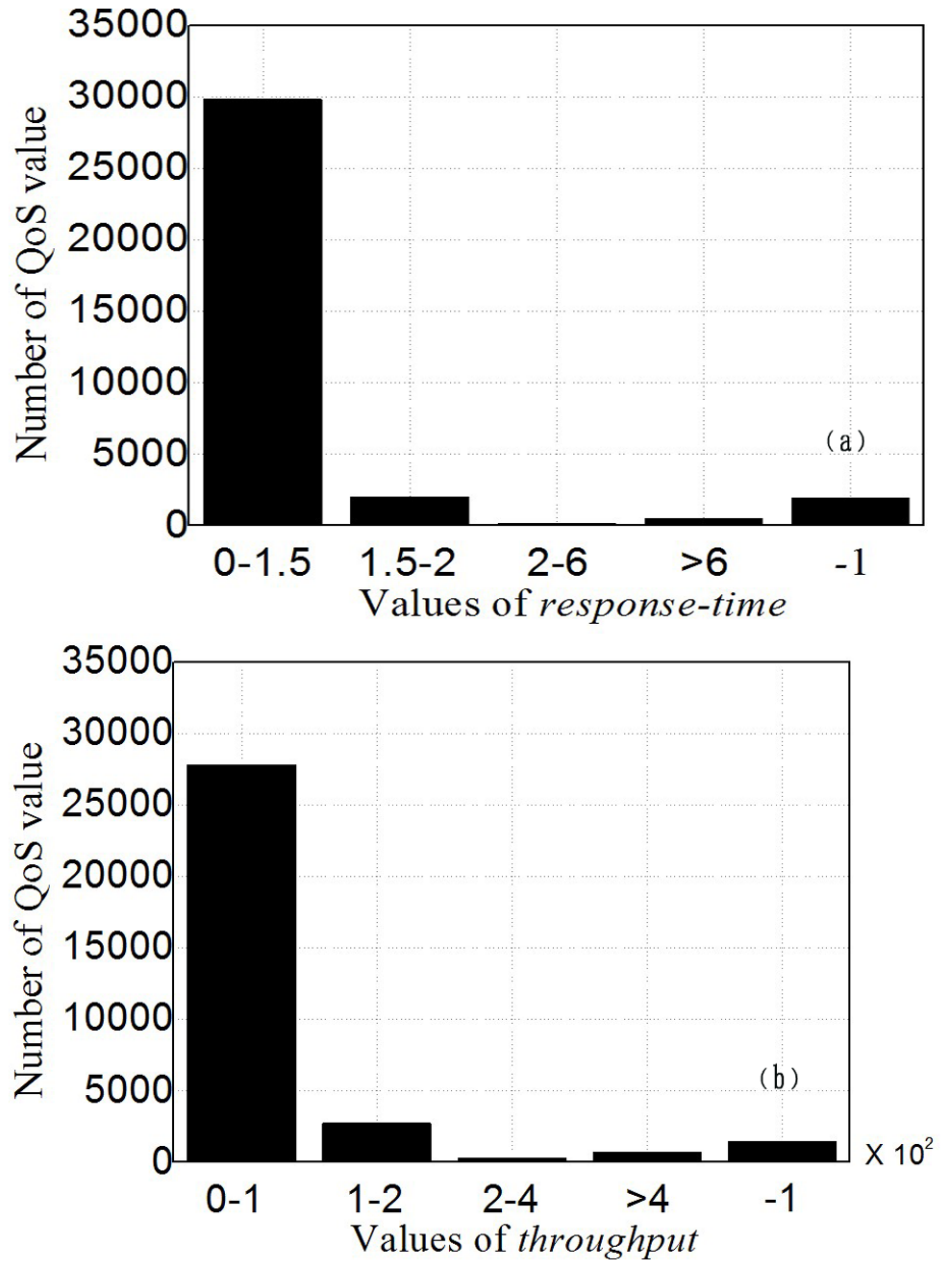
QoS value distributions. (a) and (b) depict the value distributions of *response-time* and *throughput* in our customer-service matrices, where “−1” indicates that the service invocation failed due to an http error. The ranges of *response-time* and *throughput* are 0–16.053 seconds and 0–541.546 kbps, respectively.

**Figure 3:**
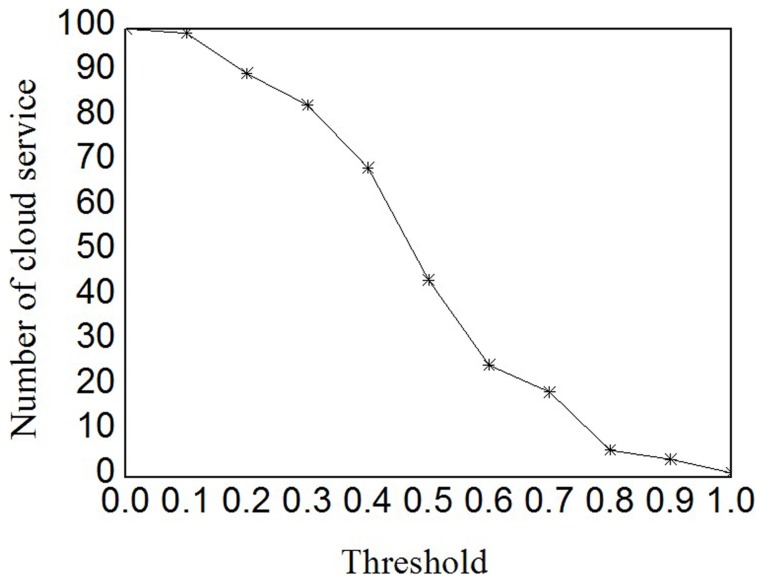
The number of recommended services for *u*_339_. Results are presented for the proposed cloud service selection approach, where the parameter 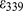 is varied from 0 to 1 in increment of 0.1.

To demonstrate the effectiveness of the proposed service selection approach, we use Microsoft C# .NET to develop a prototype system. Based on literature [8], [41], [42], we find *Availability* and *Performance* are two commonly used trustworthy attributes. We utilize them to conduct trustworthiness-aware service selection, by including two types of historical records: *response-time* and *throughput*. Their evaluation styles and weights are summarized as Table 8. We employ an open QoS research dataset [43] to simulate the historical records of Performance and Availability in cloud service market. The QoS values for *response-time* and *throughput* were collected from 339 users over 5825 web services in a real-world environment. Since it is impractical to discover and distinguish all functionally-equivalent services at the selection time, we randomly select 100 services' QoS records, and construct two 339×100 customer-service matrices for our experiment. Figure 2 shows the value distribution of *response-time* and *throughput* in *user-service* matrix. We cannot simply utilize these QoS records to analyze and rank the cloud services since these customer-service matrices are sparse assessment datasets, and cannot accurately interpret the trustworthiness status of all services. Suppose *u*_339_ is the active customer. The historical records made by *u*_339_ contains 9 and 7 missing values (on *response-time* and *throughput*, respectively) which will potentially affect his cloud service selection decision. Therefore, the proposed CF approach is employed to predict the missing attribute values. At this simulation experiment, the similarity parameter *α* is set to 0.8 and remains so until the trust values for *u*_339_ are reported. Once the prototype system obtains the customer satisfactions by utilizing Eqs.(12)–(14), where the parameter *δ = *2 and the expectation *H* ^exp^ = 0.7, the active customer will receive the trust values of each service. We vary the selection parameter 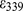 from 0 to 1 in increment of 0.1, and count the cloud services whose trust values surpass 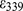 (the number of recommended services for *u*_339_, i.e. |*CS* ^339^|). The experiment results are shown in Figure 3. Although we only study two trustworthy attributes in the experiment, the proposed approach can be easily extended to other trustworthiness-aware service selection problems. When selecting the optimal trustworthy services from a set of functionally-equivalent candidates, the entry data of our approach are the corresponding historical records (i.e., QoS values or customer ratings), the active customer's preference and expectation towards service trustworthiness, and the selection parameter.

**Table 8:** Trustworthy attributes of cloud service.

Attribute	*A_j_*	Evaluation style	Weight	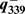	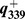
Availability	*A*_1_	“cost” QoS value	0.65	0	16.053
Performance	*A*_2_	“benefit” QoS value	0.35	0	541.546

### Impact of *δ* and *T* ^exp^

**Figure 4:**
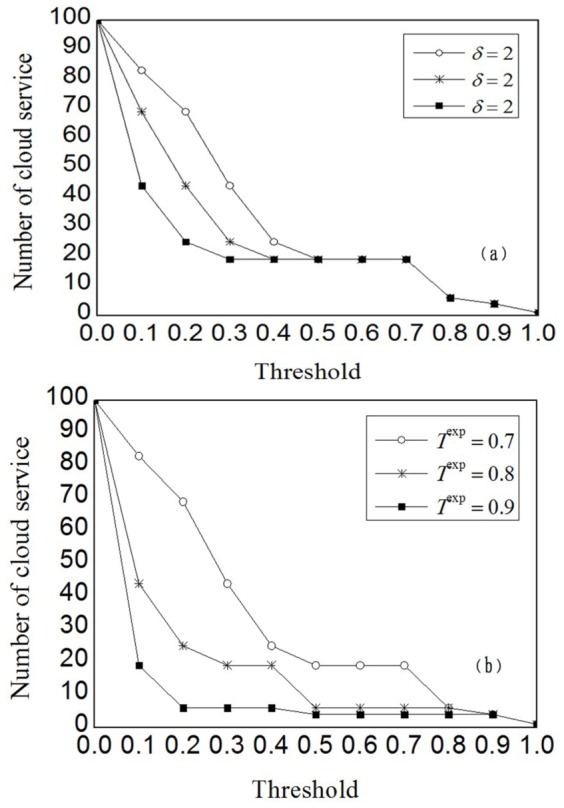
Impact of preference and expectation. (a) and (b) depict the experimental results of preference parameter *δ* and expectation *H* ^exp^, respectively. They indicates that *δ* regulates the elimination rate of untrustworthy cloud services, whereas *H* ^exp^ controls the degree of customer's tolerance to untrustworthy service.

Different customers have different preference and expectation in trustworthy service selections. Instead of risk-neutral attribute utility, we use the customer satisfaction *C_m,n_* to identify the perceived trustworthiness of delivered services. To evaluate the impact of customer's preference and expectation, we have conduct additional experiments with variable parameters *δ* and *H* ^exp^. In these experiments, we first vary *δ* from 2 to 6 in increment of 2, where the expectation *H* ^exp^ is fixed at 0.7 first. Later, we set *δ* to 2, and vary *H* ^exp^ from 0.7 to 0.9 in increment of 0.1. Figure 4 (a) shows the experimental results of preference parameter *δ* and Figure 4 (b) shows the experimental results of expectation *H* ^exp^. The parameters *δ* and *H* ^exp^ jointly determine how to derive the customer satisfaction from attribute utility to approximate the active customer's attitude towards profit and risk.

### Performance comparison of CF approaches

In this work, we present an enhanced item-based CF approach (i.e., JV-PCC) to predict the missing attribute values for cloud service selection. Our approach engages the significances (J_*n,y*_ and *VD_n,y_*) to improve the accuracy of similarity estimation. To study the prediction performance, we compare JV-PCC with two existing item-based approaches: Item-based CF adopting PCC (IPCC) [44], and Extended PCC approach (f-PCC) [31].

#### Evaluation metric

We use Mean Absolute Error (MAE) and Root Mean Square Error (RMSE) to evaluate the prediction performance of our approach in comparison with other approaches. MAE and RMSE are defined as:
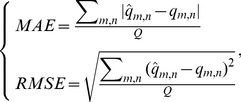
(22)where 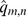 and 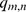 are the predicted QoS value and the actual value, respectively.

#### Experimental setup and results

**Figure 5:**
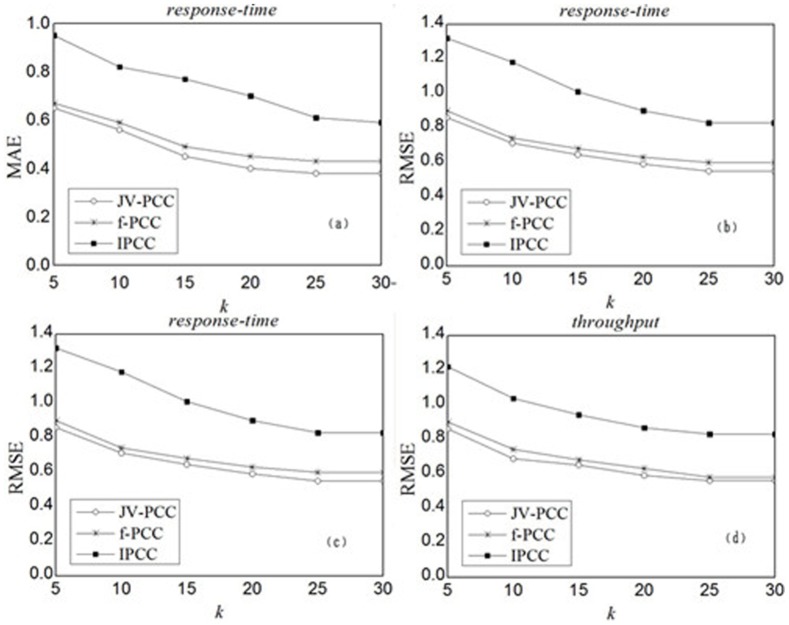
Impact of neighborhood size *k*. (a) and (b) depict the MAE fractions of JV-PCC, f-PCC and IPCC for *response-time* and *throughput*, while (c) and (d) depict the RMSE fractions. It can be observed that JV-PCC achieves smaller MAE and RMSE consistently than f-PCC for both *response-time* and *throughput*. Regardless of JV-PCC or f-PCC, as *k* increases, MAE and RMSE drop at first, indicating that better performance can be achieved by employing more similar services' records to generate the predictions. However, when *k* surpasses a specific level (i.e. *k = *25), they fail to drop with a further increase in *k*, which were caused by the limited number of similar neighbors.

The size of top-*k* similar service set plays an important role in CF approach, which determines how many neighbors' historical records are employed to generate predictions. To study the impact of neighborhood size *k*, we separate the customer-service matrices into two parts: training set (80% historical records in the matrix) and test set (the remaining 20% records). We set the density to 50%, the significance parameter *α* to 0.7, and vary *k* from 5 to 30 in increment of 5. Figure 5 shows the experimental results for *response-time* and *throughput*. Under the same simulation condition, JV-PCC and f-PCC significantly outperform IPCC. The observations also suggest that better accuracy can be achieved by our model when more historical records are available in the service selection study.

## Conclusions

Trustworthiness-aware service selection is a critical issue among cloud computing and service-oriented architecture communities. In this paper, we propose a personalized service selection approach which takes into account the missing value prediction and the multi-attribute evaluation requirements. We find that the proposed approach can tackle various types of trustworthiness-aware selection problems in cloud service market. Meanwhile, the experimental results demonstrate that the proposed CF approach significantly improves the prediction performance as compared with other competing item-based approaches.

Employing untrustworthy cloud service will expose users to high-risk IT structure, resulting in a host of intra-organizational hazards that detriment the organization and disrupt the normal operations [45]. In the present work, we can only look into the static approach for trustworthy service selection, and we will investigate more types of trustworthiness evaluation models (e.g. probability model, dynamic model, etc) in the future since different cloud service applications may have different selection criteria and data structures.
