# Correction: Decision Support for Personalized Cloud Service Selection through Multi-Attribute Trustworthiness Evaluation

**DOI:** 10.1371/journal.pone.0107821

**Published:** 2014-09-03

**Authors:** The PLOS ONE Staff

The second author’s name is misspelled. The correct name is: Cheng-Yi Xia.
